# Association of heat-shock protein 70.1 gene with physiological and physical performance of Bali cattle

**DOI:** 10.14202/vetworld.2024.17-25

**Published:** 2024-01-04

**Authors:** Ikhsan Suhendro, Ronny Rachman Noor, Jakaria Jakaria, Rudy Priyanto, Wasmen Manalu, Göran Andersson

**Affiliations:** 1 Department of Animal Production and Technology, Faculty of Animal Science, IPB University, Bogor 16680, Indonesia; 2 Department of Animal Science, Tulang Bawang University, Bandar Lampung 35121, Indonesia; 3 Department of Anatomy, Physiology, and Pharmacology, School of Veterinary Medicine and Biomedical Science, IPB University, Bogor 16680, Indonesia; 4 Department of Animal Breeding and Genetics, Swedish University of Agricultural Sciences, Uppsala 75007, Sweden

**Keywords:** Bali cattle, gene expression, heat stress, *HSP70*, polymorphism, single-nucleotide polymorphism

## Abstract

**Background and Aim:**

Global warming challenges cattle productivity and welfare since it affects heat stress and scarce feed. The heat-shock protein 70 (*HSP70*) gene is essential in cytoprotection against stressors, protecting cells from dysregulated gene expression and apoptosis. This study aimed to identify significant genetic markers of the *HSP70.1* gene that can be leveraged genetically to enhance thermotolerance and production in Bali cattle further.

**Materials and: Methods:**

Animals were sampled from three different rearing systems. In this study, 83 healthy adult male Bali cattle without abnormalities were utilized. Single-nucleotide polymorphism (SNP) diversity associated with the physiological and physical traits of Bali cattle was assessed using SNPStat online software. Gene expression for putative SNPs and their genotypic groups was further evaluated.

**Results:**

There were 15 polymorphic SNPs (c.-185G>A, c.-69T>G, c.10G>C, c.19A>G, c.45C>T, c.101INS, c.115T>C, c.130T>C, c.136G>T, c.159G>C, c.164G>T, c.234G>A, c.303G>A, c.333C>A, and c.456C>T) identified, of which 12 were associated with the assessed trait. Nine SNPs were associated with physiological traits, while eight were with physical traits. The c.136G>T as a novel, high minor allele frequency, and associative SNP was selected for *HSP70* gene expression. Individuals with the TT genotype have a trim physique, susceptible physiology, and high *HSP70* mRNA expression. On the other hand, the GG genotype was significantly associated with larger physique, lower physiology, and low *HSP70* mRNA expression. The higher expression may indicate that *HSP70.1* is involved in mitigating the deleterious effects of stress. As a result, the animal experienced negative energy balance, decreasing body size.

**Conclusion:**

Single-nucleotide polymorphism c.136G>T is a candidate biomarker for heat resistance traits in Bali cattle.

## Introduction

Animals are constantly exposed to short- and long-term environmental changes due to temperature, geographical location, nutrition, and human disturbances, which negatively affect their resilience. Heat stress and nutritional deficiency are the most critical distressing factors in animal welfare, health, production, and reproduction worldwide, especially in tropical regions [1]. Climate change is exacerbated by increasing ambient temperature and feed scarcity [2]. As a result, the production potential is jeopardized, thereby affecting sustainability and financial burden. Cattle suffer numerous issues related to productivity, reproduction, health, and mortality due to heat stress exposures [3]. Individuals subjected to heat stress must dissipate excess heat loads through conduction, convection, and evaporation [4], which exposes livestock to multiple stressors in combination with lack of nutrition. Animals have adapted well to a single stressor without altering normal body functions. However, when faced with cumulative stress with more than single stressors [1], they simultaneously had a detrimental effect on growth and reproduction. Their energy reserves are insufficient to effectively cope with these multiple stressors, impairing their ability to adapt and maintain normal homeostatic physiology [5].

Genetic and genomic selection for increased heat resistance or physical stability may improve resiliency and animal welfare [6] because within-breed genetic heterogeneity is associated with thermotolerance and animal production [7]. Heat resistance is a polygenic multifactorial trait influenced by genetic and epigenetic factors. Heat-shock protein 70 (*HSP70*) is one of the major genes widely used as a biomarker for heat stress. Heat-shock proteins are encoded by a conserved multigene family and are found in almost all organisms [8]. These proteins function as chaperones to prevent misfolding and denaturation due to heat stress [8]. The *HSP70.1* gene (other designations: *HSPA1A* or *HSPA1*) located on chromosome 23q13 in the cattle genome encodes the most well-known HSP and is widely studied for its role in heat resistance in livestock.

Genetic polymorphisms and expression levels of the *HSP70.1* gene in indigenous tropical cattle in Bali, Indonesia, and their association with physical and physiological parameters under heat stress conditions are unknown. Therefore, this study investigated the natural responses of physical and physiological traits during heat stress and nutritional shortage in Bali cattle and their association with genetic polymorphisms and *HSP70.1* mRNA expression. The ultimate goal was to identify significant genetic markers that can be genetically leveraged to enhance thermotolerance and production in Bali cattle.

## Materials and Methods

### Ethical approval

This study was approved by Animal Ethics Committee of Udayana University, Denpasar, Indonesia (Code ID: B/184/un14.2.9/pt.01.04/2021).

### Study period and location

The study was conducted from June to December 2021 in the Center of Superior Cattle Breeding and Forage (BPTU-HPT) Denpasar, Bali (8°25’3” South Latitude, 114°51’49” East Longitude, altitude 46 m) and the Center of Cattle Breeding and Forage (BPT-HMT) Serading, West Nusa Tenggara (8°34’04” South, 117°29’48” East Longitude, altitude 50 m).

### Animal and sample selection

A total of 82 healthy adult Bali bulls that reached sexual maturation (>2 years of age). They included 52 heads from Bali and 30 heads from Serading. Serading animals were exposed to heat stress and feed restriction (HS/RF). In Bali, animals were divided into two categories: bulls exposed to heat stress and well-feeding (HS/WF), and bulls housed at neutral temperature and well-feeding (TN/WF).

### Measurement of physiological and physical traits

Physiological traits, including respiration rate (RR), heart rate (HR), (rectal temperature [TR] in °C), and Benezra’s coefficient of adaptability (BCA), were evaluated as indicators of the heat stress response [9]. Body weight (BW), body length (BL), wither height (WH), chest circumference (CC), hair length (HL), body darkness (BD), and body condition score (BCS) were also measured for physical performance. Measurements were performed in a handling cage in the morning and afternoon.

### Extraction, polymerase chain reaction (PCR) amplification, and sequencing

The jugular vein was targeted to collect up to 12 mL of blood per sample, which was separated into two tubes: A 10 mL ethylenediaminetetraacetic acid tube for DNA analysis and a 2 mL tube for mRNA analysis with 1.5 mL + 0.5 mL RNA shield. The samples were then stored at 20°C in a refrigerator until use. DNA was extracted from whole blood using a DNA extraction kit from Geneaid® (Geneaid Biotech Ltd., Taiwan) and a pair of primers (Table-1), which were manually designed in Primer3 [10] and evaluated using Primer Stat.

**Table-1:** Primers used for *HSP70.1* gene target amplification.

GenBank	Primer sequence	GC %	Product	Ta
*HSP70.1*, *Bovine* (AY149618.1)	F: CCCATTACCCCTTTCCGAGA	55	714 bp	67°C
	R: TTAGGCTTGTCTCCGTCGTT	50		

GC%=Composition of guanine and cytosine in the primer, bp=Base pairs, Ta=Annealing temperature, *HSP70.1=*Heat-shock protein 70

DNA amplification was performed using a Master Cycler Gradient machine (ESCO, Singapore) using the PCR method Each reaction was performed at a final volume of 15 μL with a composition of 1 μL sample DNA, 6.1 μL NFW, 0.2 μL forward primer, 0.2 μL reverse primer, and 7.5 μL MyTaq^®^ Redmix. (Meridian, Ohio, US) The reaction conditions were denaturation at 95°C for 5 min, 94°C for 10 s, annealing at 67°C for 20 s, extension at 72°C for 30 s, and final extension at 72°C for 5 min. The number of cycles was 35. The amplification product was then visualized in a 1.5% agarose gel stained with FloroSafe DNA and photographed using a UV transilluminator (Bio-Rad, Hercules, CA, USA). Finally, the amplification product was sequenced using the ABI® PRISM big dye kitv3.1 by First BASE Laboratory (Selangor, Malaysia).

### *HSP70* diversity analysis

The nucleotide sequence results in a chromatogram were identified using FinchTV 1.4.0 (Geospiza, Inc.; Seattle, WA, USA; www.geospiza.com). Double peak bands were considered as heterozygous positions. We aligned the target gene sequence with the reference sequence (GenBank^®^: AY149618.1) using MUSCLE in MEGA 11 software (https://www.megasoftware.net/), single-nucleotide polymorphisms (SNPs) genotype, and diversity reconstruction using PopGene 1.32 (https://sites.ualberta.ca/~fyeh/popgene.html).

### Single-nucleotide polymorphism and association analyses

A logistic regression model was used to analyze the association between codominant model genotype variants and phenotypic responses. In addition, we adjusted the association results for covariance factors for differences in rearing conditions. The model built in association analysis was as follows:

Y_ijk_ = μ + g_j_ + r_i_ + ϵ_ijk_,

Y_ijk_ is the observation, μ is the mean, g_j_ is the genotype effect, r_i_ is the covariant of the rearing condition, and ϵ_ijk_ is the error. Microsoft Excel (Microsoft Office, Washington, USA), SAS^®^ 9.4 (https://support.sas.com/software/94/) [11], and online software SNPStats (https://www.snpstats.net) were used to process and analyze the association and gene expression data.

### Real-time quantitative PCR and the quantification

Samples were collected from each genotype group based on SNP c.136G>T with eight individuals of each genotype and three replications. Total RNA was prepared from whole blood and preserved in RNAshiled medium (Zymo Research, CA, USA). The RNeasy Mini Kit (Qiagen, Hilden, Germany) was used to extract RNA from blood samples. Polymerase chain reaction reverse transcriptase synthesis was obtained by transcribing RNA into complementary DNA (cDNA) using First Strand cDNA, according to the manufacturer’s protocol (Thermo Scientific, Vilnius, Lithuania). The cDNA was quantified using a NanoDrop spectrophotometer (ND-1000, Thermo Scientific, Waltham, MA, USA).

The *HSP70* cDNA target sequences were amplified using an *HSP70* specific primer pair. Primers specific for housekeeping genes (*GAPDH* and *β-Actin*) were used for normalization (Table-2). *HSP70*, *GAPDH*, and *β-Actin* were used in GenBank® accession numbers U09861 [12], NM_001034034.2 [13], and NM_173979.3 [14], respectively. Quantitative real-time PCR analysis was performed using the AG qTower4 (Channel Analytic Engine, Jena, Germany) with SYBR™ Green PCR Master Mix (Invitrogen, Massachusetts, US). Relative expression levels of *HSP70* mRNA were quantified using 2^-ΔΔ*CT*^ calculations, which were corrected and normalized using housekeeping genes [15].

**Table-2:** Primer pairs for gene mRNA amplification in qRT-PCR.

Target	Primer sequence	Product	Ta (°C)
*HSP70*, Bovine (U09861)	F: TACGTGGCCTTCACCGATAC	171 bp	57
	R: GTCGTTGATGACGCGGAAAG		
GADPH, Bovine (NM_001034034.2)	F: CCAACGTGTCTGTTGTGGATCTGA	218 bp	57
	R: GAGCTTGACAAAGTGGTCGTTGAG		
β-Actin, Bovine (NM_173979.3)	F: AGGCATCCTGACCCTCAAGTA	95 bp	57
	R: GCTCGTTGTAGAAGGTGTGGT		

*HSP70=*Heat-shock protein, GADPH=Glyceraldehyde 3-phosphate dehydrogenase, bp=Base pairs, Ta=Annealing temperature, qRT-PCR=Quantitative real time-polymerase chain reaction

## Results

### Microclimate and feed environment condition

We monitored the microclimate information in the experimental rearing system, as shown in Table-3. Temperature neutral and well feed (TNWF) was mild temperature humidity indices (THI) (<77), whereas heat stress and well feed (HSWF) and heat stress and restricted feed (HSRF) had moderate to severe heat stress (>80) [16]. Rearing locations in HSWF and HSRF were on pastures where cattle were exposed to direct heat stress. Therefore, shading in TNWF can reduce the negative impact of heat stress because it reduces direct solar exposure [17]. Shade significantly reduces the RR, body temperature, and panting score in calves at risk of heat stress; even cattle prefer shade over sprinklers when exposed to heat stress, both behaviorally and physically [17].

**Table-3:** Microclimatic conditions and daily feeding of Bali cattle in different rearing systems.

Parameter	HSRF		HSWF		TNWF	
		
	Morning	Noon	Morning	Noon	Morning	Noon
Ambient temperature (°C)	28.37	34.10	27.02	30.89	23.97	26.70
Relative humidity (%)	58.33	40.33	65.22	54.55	89.67	77.50
THI	76.51	81.45	76.22	80.04	74.14	77.26
Shade	NA		NA		Exist	
Water	*Ad libitum*		*Ad libitum*		*Ad libitum*	
Forage (%BW)	5%		8% + roughage		10%	
Concentrate (%BW)	-		-		1–1.5%	

HSRF=Heat stress restricted feed, HSWF=Heat stress well feed, TNWF=Temperature neutral well feed, Ta=Ambient air temperature, RH=Relative Humidity, THI=Temperature humidity index, BW=Body weight, SE=Standard error, CV=Coefficient of variance, NA=Not available

Feeding variables are designed to assess variations in the response of livestock to multiple stresses. Forage should be provided at 10% of the animal’s BW to obtain an average daily weight gain of 0.320 to 0.810 kg/head/day for Bali cattle [18]. TNWF and HSWF feedings were adequate for the growth of the Bali cattle. However, feeding only once a day and a small amount of HSRF is considered insufficient (Table-4).

**Table-4:** Nutritional composition of cattle feeding in each rearing system.

Condition	Feeding	Feed type	Feed name	Feed composition (%)				

				DM	CP	CF	EE	Ash
TNWF	1.25% BW	Consent.	NC64 Charoen P.	13.00	19.00	12.00	5.00	10.00
	10% BW	Forage	withered elephant grass	19.90	10.20	34.20	1.60	11.70
HSWF	8% BW	Forage	withered elephant grass	19.90	10.20	34.20	1.60	11.70
	ad-lib	Roughage	*Paspalum notatum*	24.70	14.20	31.50	2.40	10.70
	ad-lib	Roughage	*Brachiaria decumbent*	26.80	8.90	31.40	1.90	8.60
HSRF	5% BW	Forage	King grass	22.40	13.50	34.10	1.70	18.15
		Forage	*Leucaena leucocephala*	29.90	23.30	20.00	4.00	8.50
		Forage	Corn straw	55.07	9.00	27.38	2.90	7.00
		Forage	*Gliricidia sepium*	25.30	22.30	19.70	4.20	10.00
		Forage	Star grass	30.60	9.90	37.00	1.60	7.30

HSRF=Heat stress restricted feed, HSWF=Heat stress well feed, TNWF=Temperature neutral well feed, BW=Body weight, DM=Dry mater, CP=Crude protein, CF=Crude fiber, EE=Ether extract

### Heat stress effects on physiology and physics traits of Bali cattle

The rearing system significantly affected the physiological and physical performance of Bali cattle (p < 0.05) (Table-5). The physiological responses of RR, HR, and TR were significantly increased under heat stress compared to the TNWF condition. Simultaneously, heat tolerance coefficient, Benezra’s coefficient of adaptability (BCA), and dairy search index showed a significant increase in the heat tolerance index. The physical traits of BW, WH, BL, CC, BGL, BCS, BD, and ST were significantly higher on TNWF than on HSRF and HSWF. Simultaneously, the HL was significantly shorter in the TNWF condition. These physiological and physical traits change in response to heat stress, indicating that animals adapt to environmental conditions.

**Table-5:** The response of the physiology and physical traits of Bali cattle in different systems of heat stress conditions.

Parameters	HSRF	HSWF	TNWF	Mean	SE	CV	p*-*Value
Physiologies							
RR (beats/min)	30.10^a^	34.19^a^	25.40^b^	30.55	0.58	24.11	***
HR (beats/min)	82.43^a^	62.88^b^	53.02^c^	68.27	1.50	27.91	*
TR (°C)	38.77^b^	38.93^a^	38.09^c^	38.68	0.04	1.32	***
HTC	95.33^b^	93.70^c^	102.08^a^	96.2	0.40	5.30	***
BCA	2.05^b^	2.20^a^	1.86^c^	2.07	0.02	12.59	***
DSI	1.10^a^	1.09^a^	1.03^b^	1.08	0.01	9.07	*
Physics							
BW (kg)	141.48^c^	180.95^b^	251.39^a^	184.74	6.49	32.53	***
WH (cm)	94.50^c^	109.72^b^	114.88^a^	105.26	1.27	10.98	**
BL (cm)	99.50^c^	105.52^b^	113.74^a^	105.42	0.96	8.28	***
CC (cm)	132.70^c^	139.76^b^	155.21^a^	141.20	2.39	15.20	***
BGL (mg/dL)	49.42^b^	53.63^b^	61.95^a^	54.39	1.32	20.96	***
BCS (fat=4)	2.03^c^	3.27^b^	3.83^a^	2.96	0.09	28.89	***
BD (dark=4)	1.93^c^	3.06^b^	3.95^a^	2.16	0.13	41.09	***
ST (mm)	6.63^b^	15.16^a^	15.13^a^	11.91	0.22	39.94	***
HL (mm)	5.59^a^	3.41^b^	2.60^c^	4.20	0.54	40.37	***
SC (cm)	22.48	23.97		23.29	0.84	27.56	0.3798

HSRF=Heat stress restricted feed, HSWF=Heat stress well feed, TNWF=Temperature neutral well feed, different superscripts in the same column denote a significant difference, *, **, *** are significance values at 0.05, 0.01, and 0.001, respectively

### Single-nucleotide polymorphisms in partial amplification of the *HSP70.1* gene

**Figure-1:**
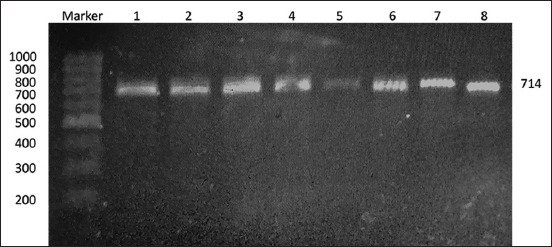
Polymerase chain reaction results in the *heat-shock protein 70.1* gene. Marker using 100 bp ladder size. Total target amplification along 714 bp. bp=Base pairs.

**Figure-2:**
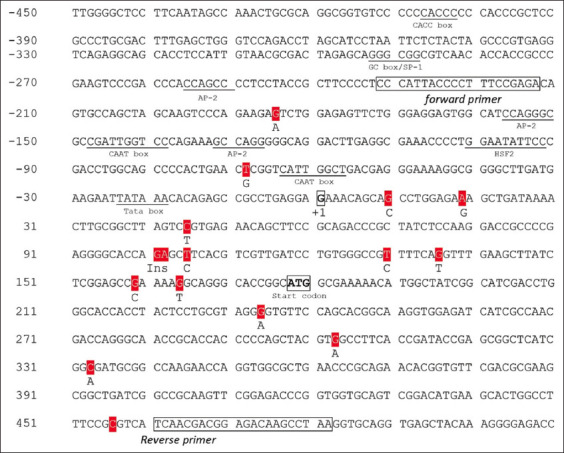
The nucleotide sequence of the *HSP70.1* gene and its target fragment. The target amplification was obtained from the primer pair in block notation. Red highlights indicate mutation points, and underlines indicate transcription factor binding sites. The transcription site (+1) denotes the start of the 5’-UTR, and the start codon (ATG) starts at the +178^th^ bp. *HSP70.1=*Heat-shock protein 70, UTR=Untranslated region, bp=Base pairs.

**Figure-3:**
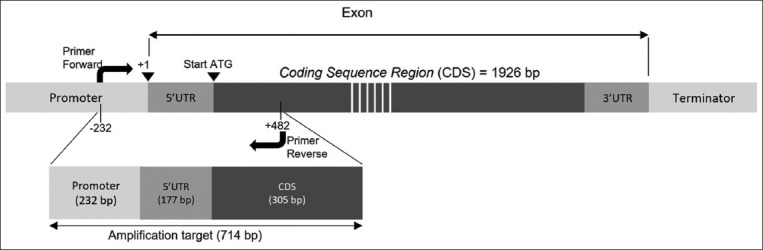
Diagrammatic representation of the amplification target of the *HSP70.1* gene in Bali cattle. The *HSP70.1* gene has a total exon length of 1926 bp. The target amplification is 714 bp in the 5’ UTR and partial of the promoter and CDS. *HSP70.1=*Heat-shock protein 70, UTR=Untranslated region, CDS=Coding sequence, bp=Base pairs.

Partial amplification of the *HSP70.1* gene in Bali cattle was amplified and sequenced. The product target amplification along 714 bp (Figure-1) consists of 232 bp 5′ flanking regions, 177 bp 5′ UTR, and 305 coding sequence. Figures-2 and 3 present the *HSP70.1* sequence target, SNPs, and nucleotide position.

Table-6 lists the gene, location, dbSNP, and allele frequency information of the SNPs. Only SNPs with a minor allele frequency (MAF) of more than 5% were used for analysis. A total of 15 SNPs were identified, of which 69, +19, +45, and +101 were reported in our previous study [19]. Twelve SNPs were found in the dbSNP database (https://www.ensembl.org), of which three were novel.

**Table-6:** SNP polymorphic site positions in *HSP70.1* gene in 82 heads of Bali cattle.

SNP	Position	Chr position	Accession no.	Allele frequency		AA change
c.-185G>A	Promoter	23:27522941	Novel	G: 0.88	A: 0.12	-
c.-69T>G	Promoter	23:27522825	rs797598758	T: 0.61	G: 0.39	-
c. 10G>C	5’- UTR	23:27522747	rs384316213	G: 0.95	C: 0.05	-
c. 19A>G	5’- UTR	23:27522738	rs463165186	A: 0.52	G: 0.48	-
c. 45C>T	5’- UTR	23:27522712	rs211506802	C: 0.96	T: 0.04	-
c. 101INS	5’- UTR	23:27522655	Novel	-:0.85	i: 0.15	-
c. 115T>C	5’- UTR	23:27522653	rs450184276	T: 0.82	C: 0.18	-
c. 130T>C	5’- UTR	23:27522627	rs443089752	T: 0.91	C: 0.09	-
c. 136G>T	5’- UTR	23:27522621	Novel	G: 0.69	T: 0.31	-
c. 159G>C	5’- UTR	23:27522597	rs209592471	G: 0.84	C: 0.16	-
c. 164G>T	5’- UTR	23:27522593	rs208480184	G: 0.80	T: 0.20	-
c. 234G>A	CDS	23:27522523	rs384482068	G: 0.82	A: 0.18	Gly/Gly
c. 303G>A	CDS	23:27522454	rs135145204	G: 0.83	A: 0.17	Val/Val
c. 333C>A	CDS	23:27522424	rs110903970	C: 0.76	A: 0.24	Gly/Gly
c. 456C>T	CDS	23:27522301	rs480841468	C: 0.55	T: 0.45	Arg/Arg

The transcription initiation site is+1, AA=Amino acid changes, *HSP70.1=*Heat-shock protein 70, SNP=Single-nucleotide polymorphism

Five SNPs, c.-185G>A, c.10G>C, c.136G>T, c.159G>C, and c.333C>A, were associated (p < 0.05) with both physiological and physical traits (Table-7). Individuals with mutant SNP genotypes of -185, +136, +159, +164, +234, and +303 had smaller physical measurements than those with the other genotypes. Furthermore, individuals with the mutant genotype of SNPs at positions -185, +45, +115, +130, +136, and +159 had higher physiological responses (p < 0.05) than individuals carrying other genotypes. Novel SNPs of -185 and +136 could be biomarkers because they were associated with excellent stress response traits.

**Table-7:** Significant association of SNPs in the *HSP70* gene on physiology and physical traits in Bali cattle.

SNP	Traits	Category	Genotype			Mean	SE

			A/A	A/B	B/B		
c.-185G>A	DSI	Physiology	1.08^a^	1.06^a^	0.97^b^	1.07	0.01
	RRm	Physiology	27.52^c^	28.73^b^	38.67^a^	28.21	0.70
	BCAm	Physiology	2.21^b^	2.26^b^	2.69^a^	2.24	0.03
	BW	Physic	188.68^a^	175.16^b^	110.44^c^	182.95	7.00
	BCS	Physic	3.17^a^	2.91^b^	2.00^c^	3.07	0.11
	ST	Physic	12.80^a^	11.68^b^	7.57^c^	12.36	0.58
c. 10G>C	BCAm	Physiology	2.22^b^	2.40^a^	NA	2.24	0.03
	ST	Physic	12.31^b^	12.86^a^	NA	12.36	0.58
c. 45C>T	RRm	Physiology	27.71^b^	33.33^a^	NA	28.21	0.70
	BCAm	Physiology	2.21^b^	2.46^a^	NA	2.24	0.03
c. 115T>C	RRm	Physiology	27.60^c^	28.95^b^	34.00^a^	28.21	0.70
	DSI	Physiology	1.09^a^	1.04^b^	1.03^b^	1.07	0.01
c. 130T>C	BCAn	Physiology	2.46^a^	2.31^b^	2.31^b^	2.44	0.05
	HRm	Physiology	65.57^b^	56.00^c^	73.00^a^	64.66	2.18
c. 136G>T	BCAn	Physiology	2.47^a^	2.32^b^	2.52^a^	2.44	0.05
	HRm	Physiology	62.76^b^	63.72^b^	72.08^a^	64.66	2.18
	BW	Physic	191.76^a^	182.90^b^	155.87^c^	182.95	7.00
	BCS	Physic	3.11^b^	3.17^a^	2.83^c^	3.07	0.11
	CC	Physic	141.70^b^	146.64^a^	132.88^c^	141.45	2.26
c. 159G>C	HRm	Physiology	62.31^c^	67.38^b^	77.25^a^	64.66	2.18
	DSI	Physiology	1.08^a^	1.08^a^	1.00^b^	1.07	0.01
	BGL	Physic	52.96^b^	63.88^a^	59.00^c^	55.08	1.43
	CC	Physic	140.60^b^	152.12^a^	136.17^b^	141.45	2.26
c. 164G>T	CC	Physic	140.86^b^	151.10^a^	131.67^c^	141.45	2.26
c. 234G>A	BGL	Physics	53.84^b^	60.86^a^	56.80^c^	55.08	1.43
	BD	Physic	2.96^c^	3.43^a^	3.10^b^	3.03	0.14
	CC	Physic	140.61^b^	152.77^a^	137.68^b^	141.45	2.26
c. 303G>A	BW	Physic	184.38^a^	187.51^a^	134.83^b^	182.95	7.00
	CC	Physic	139.54^b^	149.27^a^	126.97^c^	141.45	2.26
c. 333C>A	HRm	Physiology	66.80^a^	62.14^b^	57.89^c^	64.66	2.18
	BD	Physic	2.91^c^	3.21^b^	3.33^a^	3.03	0.14
	CC	Physic	139.57^c^	147.49^a^	141.20^b^	141.45	2.26
c. 456C>T	HRn	Physiology	68.90^b^	79.93^a^	67.43^b^	70.84	2.68
	DSI	Physiology	1.05^a^	1.12^b^	1.06^a^	1.07	0.01

A/A, A/B, and B/B denote the wild type, heterozygote, and mutant genotype, SE=Standard error, NA=Not available, lowercase of m and n in the trait’s column denotes the morning and noon time, respectively, different superscripts in the same column denote a significant difference between genotypes (p *< 0*.05). *HSP70.1=*Heat-shock protein 70, SNP=Single-nucleotide polymorphism

### Gene expression levels of potential SNP c.136G>T

**Figure-4:**
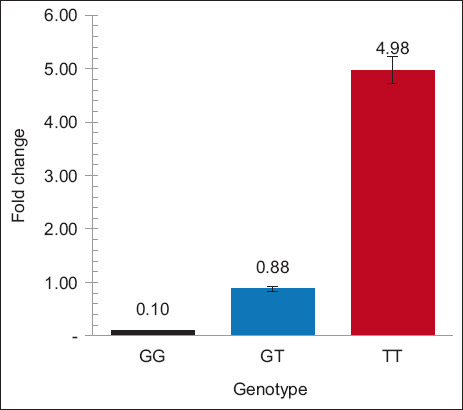
The relative expression level of *HSP70.1* mRNA in Bali cattle with genotypes variant of c.136G>T for heat tolerance traits. Relative mRNA expression using qRT-PCR technique. Significant differences (p < 0.05) between genotypes are denoted by superscript letters (^a,b,c^). *HSP70.1=*Heat-shock protein 70, qRT-PCR=Qualitative reverse transcription polymerase chain reaction.

The novel gene variant of c.136G>T was evaluated for its potential influence on differential gene expression levels due to its novel SNP, high MAF (0.31), and association with physiological and physical traits. *HSP70.1* gene expression levels in blood cells were determined for these three genotypes (Figure-4). Individuals with the TT mutant genotype had a significant (p < 0.05) trim physique, susceptible physiology, and high *HSP70* mRNA expression. On the other hand, the GG genotype was significantly (p < 0.05) associated with a bigger physique, lower physiology, and low *HSP70.1* mRNA expression.

## Discussion

Temperature humidity indices are widely used to determine heat stress levels in animals. When the ambient THI exceeds 80, there is a hazard of heat stress in animals [16]. Cattle need 10% feed of their BW to grow normally; hence, any feed shortage will have negative effects [18]. Therefore, high THI levels and insufficient nutrition are detrimental to cattle. In this study, HS/RF and HS/WF Bali cattle had an average THI of more than 80, whereas TN/WF Bali cattle were kept in temperatures ranging from comfortable to alert (<77). Therefore, in this study, the TN/WF rearing system with proper microclimates and sufficient feed is the most pleasant method for rearing animals, followed by HS/WF with good feed conditions. Excessive HS/RF was most distressing, with multiple feeding stresses insufficient and THI exceeding. Animals have developed coping mechanisms to minimize the impact of these environmental stressors on their biological systems through acclimation, acclimatization, and adaptation [20].

Breathing rate (RR), HR, and TR are the general parameters observed in bovine physiology [21]. In our study, the physiological traits RR, HR, TR, and BCA tolerance index increased significantly (p = 0.05) in HSRF and HSWF compared with those in TNWF conditions (Table-5). Under heat stress conditions, the animal’s body increases its heat dissipation capacity, manifesting as shortness of breath, rapid breath, and higher body temperature [21].

Because they were reared in an extensive pasture system, increased HS/RF and HS/RF physiology directly resulted from sun exposure. However, RR in HS/WF (34.19 beats/min) was within the standard threshold (<35 beats/min) [21]. The highest TR value of 38.93°C in HS/WF was in the standard reference threshold (<39.1°C) [21]. Therefore, when exposed to 32°C, TR and RR values should be increased beyond the standard threshold [21]. However, the physiology of Bali cattle was maintained even when reared at 35°C in HS/RF. The BCA index in this study was lower than that of taurine cattle breeds and most local tropical cattle raised under heat stress conditions [22,23]. A similar finding has also been reported for the local tropical Sahiwal cattle in India [24]. Benezra’s coefficient of adaptability is widely used to assess the heat tolerance of cattle, goats, and buffalo [9]. The lower BCA index indicates that the physiology of Bali cattle is adaptive to the THI environment but remains within the normal range of values.

Animals adapt their morphology and physiology better to dissipate heat in hot climates [4]. Bali cattle reared under TN/WF had significantly larger body dimensions, darker skin color, thicker skin, and shorter hair (p < 0.001) compared with animals reared under HS/WF and HS/RF conditions. Multiple stressors triggered by HS/RF interfered with the growth and physical traits of Bali cattle in HS/RF. Bali cattle that were reared under these conditions had much smaller body dimensions (p < 0.001). These stressors take a large part of the energy from animals that should be used for growth and reproduction instead of maintenance and survival.

Heat-shock proteins, such as *HSP70.1*, act as chaperones and prevent misfolding or denaturation [8] and are essential for stress protection. *HSP70* is widely associated with heat stress responses compared to other *HSP* gene families [8]. Therefore, identifying genetic markers, such as SNP, for selecting important traits and completing association analysis would be highly beneficial [25]. This study discovered many polymorphic SNPs (15) in the *HSP70.1* gene, which were similar to those found in local cattle in a previous Turkish and Sanhe cattle study [26, 27]. On the other hand, only a few mutations have been found in *Bos taurus* breeds [28]. Furthermore, its association with physiological and physical traits revealed the putative and versatility of this gene as a biological marker for further selection. *HSP70* has also been found as a biological marker for heat tolerance traits in other cattle breeds, such as Chinese Holstein cattle [7], Thai local cattle [29], Tharpakar, and Karan Fries [30].

**Figure-5:**
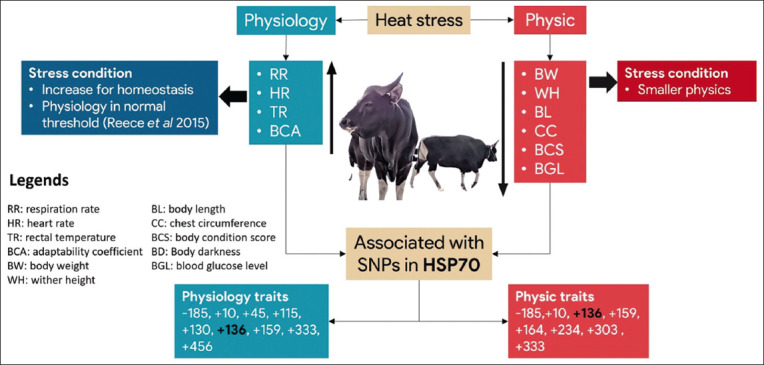
Schematic representation of the association of *HSP70.1* SNPs with physical and physiological traits of Bali cattle under heat stress conditions. Bali cattle have higher physiological and are physically smaller in heat-stress conditions. A total of 9 and 8 SNPs are associated with physiological and physical traits. These traits are associated with SNPs -185, +10, +136, +159, and +333. *HSP70.1=*Heat-shock protein 70, SNP=Single-nucleotide polymorphism [Source: Schematic design adopted from https://www.mdpi.com/2076-2615/10/11/2016].

Figure-5 shows a schematic representation of the association of SNPs in *the HSP70.1* gene with physical and physiological traits of Bali cattle under heat stress conditions. Balinese cattle are physically smaller and have higher physiological levels under heat stress. 9 and 8 SNPs were associated with physiological and physical traits, respectively. These traits were associated with SNPs -185, +10, +136, +159, and +333.

Gene expression analysis revealed the function of putative c.136G>T SNPs in the tolerance ability of Bali cattle. The GG genotype of c.136G>T had the lowest (p *=* 0.05) *HSP70.1* gene expression levels (Figure-4). These findings suggest that individuals of the GG genotype could protect their bodies from the detrimental effects of heat stress and did not require *HSP70* expression for cell repair. The lower *HSP70.1* mRNA expression may be a sign of better heat tolerance. Our findings were consistent with those of previous studies, which found that increased *HSP70* expression is due to the susceptibility of cattle to heat stress [31]. There have also been reports of increased *HSP70* expression during the induction of heat stress in Tharparkar cow [30]. Higher *HSP70* expression in susceptible animals and during heat stress suggests that *HSP70* may play a role in mitigating the adverse effects of heat stress on homeostasis and cell integrity by serving as a cell and body defense mechanism against potential heat stress-induced cell damage [8].

Interestingly, smaller Bali cattle (TT genotype of c.136G>T) had significantly higher *HSP70.1* gene expression under multiple stress conditions. This higher gene expression is likely due to the role of the *HSP70.1* gene in coping with multiple stressor perturbations. The *HSP70* gene is more expressed under heat stress conditions to protect cells from misfolding and apoptosis [8, 30]. Bali cattle have an adaptation response to lose weight due to insufficient fodder supply and low appetite caused by heat stress. This low-energy consumption is much less than required for maintenance or production, causing a negative energy balance. Negative energy balance causes adverse effects in livestock with higher periparturient disease and lower livestock performance [32].

Furthermore, *HSP70* polymorphisms associated with these physical and physiological traits demonstrated that genotypes (GG and GT) might be utilized as molecular markers for selecting heat-resistant animals. Future studies should use whole-genome markers to identify other SNPs associated with these biomarkers.

## Conclusion

Twelve of the 15 polymorphic SNPs of *the HSP70.1* gene were associated with the physiological and physical traits of Bali cattle. The novel SNPs at -185, +101, and +136 were observed. SNP +136 is a novel, high MAF, and highly associative genetic marker that can be utilized as a biomarker for heat tolerance in Bali cattle. Individuals with the GG genotype at the SNP +136 locus were thermotolerant.

## Authors’ Contributions

RRN, JJ, and IS: Study design. IS, RP, and JJ: Collected the data. IS, WM, JJ, GA, and RRN: Data analysis and interpretation. IS: Drafted the manuscript. GA and RRN: Formal analyses and drafted, critically reviewed, and revised the manuscript. All authors have read, reviewed, and approved the final manuscript.
